# Factors influencing the mental health of caregivers of children with cerebral palsy

**DOI:** 10.3389/fped.2022.920744

**Published:** 2022-11-30

**Authors:** Dania A. Kouther, Murug O. Shakir, Reem A. Alhumaidah, Huda A. Jamaluddin, Afnan Y. Jaha, Mesbah J. Alshumrani, Alqassem Y. Hakami

**Affiliations:** ^1^ College of Medicine, King Saud bin Abdulaziz University for Health Science, Jeddah, Saudi Arabia; ^2^ Research Office, King Abdullah International Medical Research Center, Jeddah, Saudi Arabia; ^3^ Department of Pediatrics, National Guard Hospital, Jeddah, Saudi Arabia

**Keywords:** cerebral palsy, caregivers, depression, anxiety, stress

## Abstract

**Objectives:**

Caregivers of children with cerebral palsy have a huge burden which might affect their mental health. This study aimed to determine the different factors affecting the mental health of caregivers of children with cerebral palsy and to raise awareness among healthcare providers.

**Methods:**

A cross-sectional study was conducted among caregivers of children with cerebral palsy in National Guard Health Affairs-Jeddah, Saudi Arabia, using the Depression Anxiety Stress Scale-21, which is a validated questionnaire that assesses: depression, anxiety, and stress. This questionnaire was used to assess the mental health of the caregivers. In addition, factors that reflected the child's health condition, such as visual impairment, number of emergency department visits, and number of Pediatric Intensive Care Unit admissions were also reported to investigate the impact on the caregiver's mental health.

**Results:**

The study sample consisted of 40 caregivers, of which 72.5% were mothers. According to the Depression Anxiety Stress Scale-21 score, 12.5% (*n* = 5) of the caregivers had moderate depression scores, 10% (*n* = 4) revealed extremely severe depression, and 10% (*n* = 4) showed moderate anxiety. Furthermore, 12.5% (*n* = 5), 15% (*n* = 6), and 7.5% (*n* = 3) of the caregivers have scored as moderate, severe, and extremely severe stress levels, respectively. Caregivers’ depression, anxiety, and stress scores were significantly (*p* ≤ 0.05) associated with the impact of vision of their dependent children, frequent hospital admissions, and frequent emergency department visits. Increased Pediatric Intensive Care Unit admissions in the past year were also significantly associated with higher caregiver anxiety scores.

**Conclusion:**

To the best of our knowledge, the dimension of caregivers’ stress and anxiety and their association with the children's dependency level is not well documented in our region. Caregivers of children with cerebral palsy reported having mental health challenges associated with the children's visual impairment, frequent need for acute medical care, and hospital admissions. Healthcare workers should provide early and proactive planning of medical and social support for children with cerebral palsy and their families using a family-centered approach.

## Introduction

Cerebral palsy (CP) refers to a heterogeneous group of permanent, non-progressive neurodevelopmental disorders varying in severity among individuals, which may manifest in early infancy or childhood. Pre-conceptional, prenatal, perinatal, and postnatal risk factors contribute to the development of cerebral palsy (1). The underlying mechanisms leading to CP include predisposing processes that may influence early brain development, such as prematurity, perinatal hypoxic-ischemic injury, and intrauterine infections (2). In addition, a two-hit model theory was linked to abnormal neurodevelopment leading to CP and it suggests that the first hit is the exposure to intrauterine insults while perinatal/neonatal complications is the second hit (2). Cerebral palsy is characterized by impaired motor function, disturbed sensations, and depressed intellectual abilities (1). Regarding the global prevalence of CP, the estimated range is between 1.5 and 4 per 1,000 live births according to population-based studies, with an overall birth prevalence of 2 per 1,000 live births (2). Moreover, the prevalence rate of CP in Saudi Arabia was estimated to be 2.34 per 1,000 Saudi children (3). Functional limitations in patients with CP may result in chronic dependency, thereby compromising caregivers’ mental health and interfering with the integrity of the family structure (4).

Concerns regarding the effects of CP on caregivers’ mental health status have been raised by healthcare providers. It has been shown that caregivers of children with CP encounter negative psychological experiences during their lifetime (5). The mental health status of primary caregivers was assessed using the General Health Questionnaire-28 (GHQ-28), a questionnaire developed by Goldberg in 1978 as a screening tool to detect those likely to have or be at risk of developing psychiatric disorders (6). The questionnaire outcomes revealed that more than half of the caregivers were susceptible to developing psychiatric disorders (5). In addition, one report demonstrated that caregivers are vulnerable to stress and cognitive and emotional problems (7). Another study indicated that the stress imposed on caregivers occurs as a consequence of the constant demand of disabled children due to limitations in communication and performance of daily tasks and self-caring activities (5). Furthermore, a study conducted in Saudi by Alzahrani et al., 2015, investigated the psychological impact of caregiving in a population with wider selection of participants. The latter study reported a total of 72.8% as prevalence of depression in caregivers of hospitalized patients (8).

A study conducted in Ontario, Canada, used the Survey Diagnostic Instrument (SDI), a 134-item questionnaire based on DSM-III criteria, and concluded that children's behavioral problems affect caregivers’ self-perception, ultimately compromising their mental health (4, 9). Additionally, caregivers’ ability to manage stress is reduced with increased behavioral problems in the child (4). Moreover, a systematic review on the impact of CP on the quality of life of the caregivers concluded that parents, particularly mothers, experience higher levels of stress and depression as compared to parents of unaffected children (10). Furthermore, this latter study demonstrated the factors consistently related with such impact, such as child behavior and cognitive problems, low caregiver self-efficacy and low social support. Additionally, a study conducted by Power et al., 2019 in Bangladesh showed that caregivers of adolescents affected by CP have markedly higher risk of depression and stress than caregivers of unaffected adolescents (Effect Size 0.1–0.2, *p* < 0.05) (11).

A cross-sectional study conducted in Bangalore, India, assessed the severity of disabilities based on the level of independence in performing daily living activities (5). Caregivers of children with moderate-to-severe disabilities had higher GHQ scores than those with milder disabilities. Furthermore, three significant negative correlations were found between the quality of life and the mental health of the primary caregivers. Family functioning, which refers to the social and structural properties of the global family environment, has been found to play a significant role in the psychological status of caregivers of children with CP (4, 12). Loss of family integrity and functioning ultimately leads to the development of adverse mental health problems in caregivers. In contrast, healthy family functioning has been reported to be associated with enhanced mental health status of caregivers of children with CP (4). While the above has been proven to affect caregivers’ mental health and impact their daily lives and relationships when studying other communities, the aforementioned factors, namely, children's behavior and cognitive problems, level of independence, caregiver self-efficacy, social support, and family functioning, have yet to be investigated in a local setting.

In this study, we investigated various factors affecting the mental health of caregivers in the Kingdom of Saudi Arabia. Moreover, we investigated the association between caregivers’ mental status and the severity and dependency of the child's condition. We also assessed the need for additional support to be provided to caregivers of children with CP by healthcare providers, governmental institutions, and society.

## Materials and methods

### Study sample

This cross-sectional study was conducted at a tertiary care hospital in the general pediatric and pediatric neurology departments of King Abdulaziz Medical City (KAMC), Jeddah, Saudi Arabia, from June 2019 to December 2021. The inclusion criteria were the following (1) Children diagnosed with CP who are older than 6 months (2) being the main caregiver of the child (based on who spends most time caring for the child). As for the exclusion criteria, caregivers previously diagnosed with psychiatric disorders were excluded from the study.

**Figure 1:**
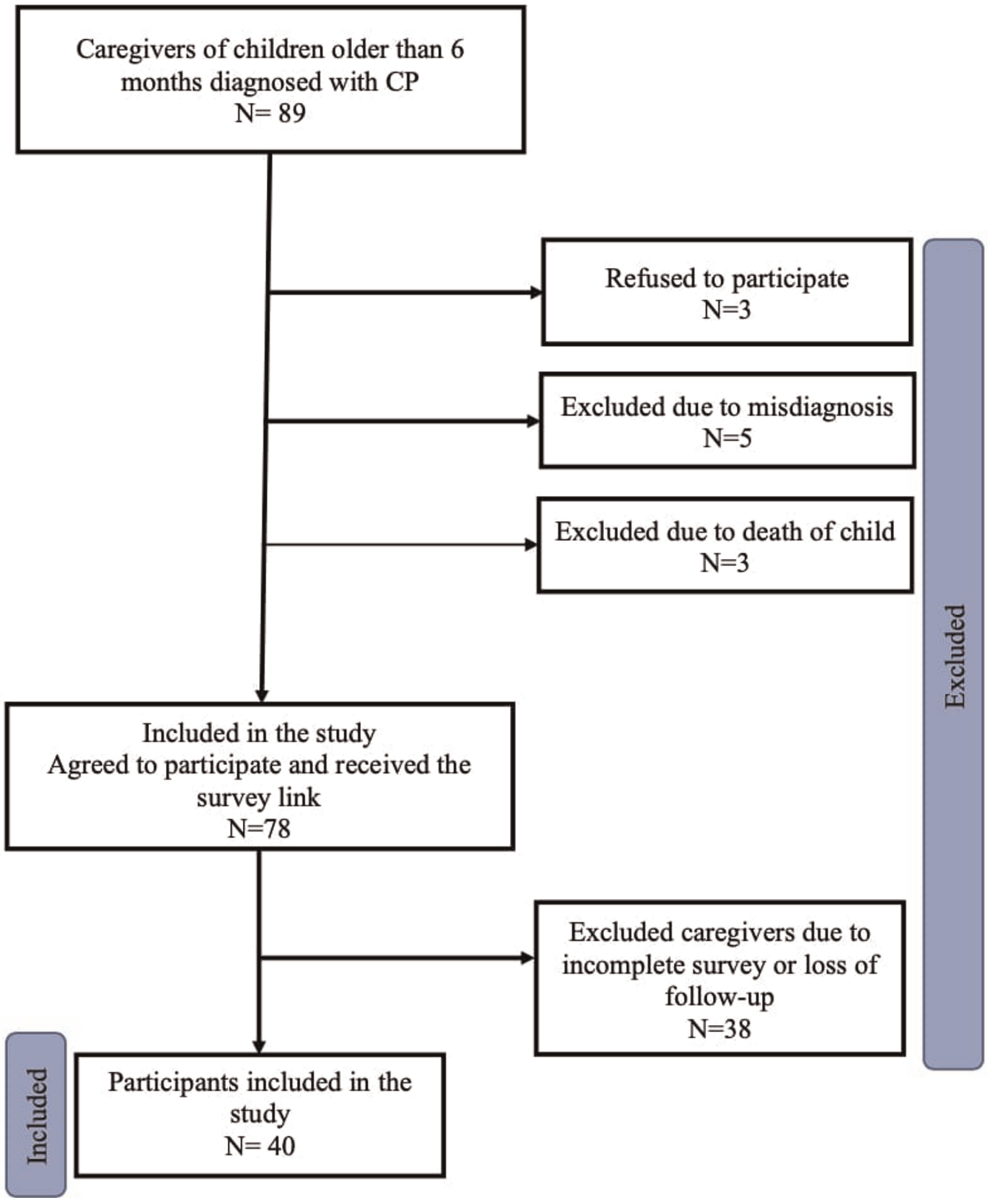
Flow chart showing the process of subjects selection. The initial sample number was 89 (Caregivers of children with CP). A total number of 75 caregivers agreed to participate in the study. However, only 40 caregivers completed the survey.

This study was conducted in a non-probability consecutive sampling technique. The MRNs of the cerebral palsy children were requested according to the Institutional Review Board (IRB) approval letter. A total of 78 primary family caregivers consented to participate in this study, of which 40 completed the questionnaire making the response rate of the study 51.3%. The study samples and the process of subject selection are illustrated in Figure 1.

Scientific approval was obtained from King Abdullah International Medical Research Center (KAIMRC), and ethical approval was obtained from the IRB (approval number SP19.125J). An explanation of the nature and aim of the study was provided to all caregivers. The DASS-21 questionnaire, a demographic information sheet, and a consent form were sent as an online link by the researchers to the primary caregivers of children with CP after obtaining verbal consent *via* a telephone call. The data of the patients and their caregivers were password-protected and only accessible by investigators to ensure patient confidentiality.

### Data collection methods

The researchers used the Depression Anxiety Stress Scale-21 (DASS-21), which was filled by the primary caregivers after obtaining their consent. The DASS-21 is a validated questionnaire designed to assess mental health by specifically measuring the magnitude of three negative emotional states: depression, anxiety, and stress (13).

The Arabic version of the questionnaire was validated for use in Arabic speaking individuals as stated in the work of Moussa et al., 2016 (14). Additionally, DASS-21 was validated and used in the Saudi population in a previous study by Almalki et al., 2021 (15). The questionnaire consisted of 21 questions divided into three subscales: depression, anxiety, and stress, each of which contained seven items. The participants were asked to rate each statement on a 4-point scale (from 0 to 3), with 0 indicating that the statement did not apply at all and 3 meaning that it applied very much or most of the time over the last week. The scores on each subscale were summed and multiplied by two before analysis. Higher scores indicated an increasing severity of depression, anxiety, or stress (13). Finally, it is important to note that this questionnaire cannot diagnose depression, anxiety, or stress. Instead, it provides an indication of whether these problems influence a person's quality of life. The questionnaire is available in both English and Arabic and takes 5–10 min to complete.

Researchers also obtained demographic data of the caregivers to assess their psychological, educational, social, and financial status. The data included age, gender, marital status, relationship to the child, and the number of other children with CP. Additionally, level of education, employment status, and income were obtained. Moreover, information about the severity and dependency of the child diagnosed with CP was also acquired. This was evaluated by asking questions about the children's age, visual/hearing impairment, feeding method, motor skills, activities of daily living, and attendance of a learning facility. Furthermore, researchers inquired about the frequency of the children's hospital admissions, PICU admissions, and ED visits in the last year. The number of specialties the child visits at the clinic and whether they receive physical/occupational therapy were also assessed.

### Statistical analysis

Excel was used for data entry and SPSS version 26 was used for data coding and analysis. To test the relationship between variables, qualitative data were expressed as numbers and percentages, and the Fisher's Exact test was applied. Quantitative data were expressed as medians and interquartile ranges (IQR) for non-normally distributed data with a confidence interval (CI) of 95%. Differences were considered statistically significant at a *p*-value of 0.05.

## Results

### Caregivers demographics

Table 1 summarizes the main demographic characteristics of the caregivers who participated in the study. The majority of the caregivers were mothers (72.5%, *n* = 29). Regarding age, 47.5% (*n* = 19) of the caregivers were aged 35–45 years. In addition, most caregivers were married (95%; *n* = 38). Regarding educational level, half of the caregivers had a bachelor's degree or diploma, 40% (*n* = 16) held a high school degree or below, and the rest had a higher education level. Moreover, 52.5% (*n* = 21) of the caregivers were unemployed mothers. Furthermore, 47.5% (*n* = 19) of caregivers had a monthly income of more than 9,000 SAR and 42.5% (*n* = 17) of caregivers had 1–3 other children with CP.

**Table 1:** Distribution of the participant parents according to their characters, overall number of children and number of those having CP.

Variable	No. (%)
***Relationship to the child***	
Father	11 (27.5)
Mother	29 (72.5)
***Caregiver's age***	
18–25	13 (32.5)
35–45	19 (47.5)
>46	8 (20)
***Marital status***	
Married	38 (95)
Divorced	2 (5)
***Level of education***	
High school degree or below	16 (40)
Bachelor's degree/Diploma	20 (50)
Higher education (Masters or PhD)	4 (10)
***Employment status***	
Employed	18 (45)
Unemployed	21 (52.5)
Retried	1 (2.5)
***Monthly income (Saudi Riyals)***	
<3,000	5 (12.5)
4,000–9,000	16 (40)
>9,000 SAR	19 (47.5)
***No. of other children with CP***	
None	20 (50)
1–3	17 (42.5)
NA	3 (7.5)

### Children demographics

The age range of 60% (*n* = 24) of children in our study was 6–15 years. Moreover, the majority (75%, *n* = 30) had intact vision. While 80% (*n* = 32) of the children were fed orally, 20% (*n* = 8) were dependent on a nasogastric tube for feeding. In addition, 35% (*n* = 14) of patients with CP in this study required a wheelchair for mobility with the assistance of caregivers, whereas 32.5% (*n* = 13) walked with difficulty. Regarding daily activities, 50% (*n* = 20) were unable to perform activities of daily living. The children's motor function was also further classified according to Gross Motor Function Classification System (GMFCS) into five levels as demonstrated in Supplementary Table A in the supplementary material.

Of the children attending a learning facility, 15% (*n* = 6) went to rehabilitation centers and 17% (*n* = 7) went to school. A total of 27.5% (*n* = 11) of the children attended these facilities daily.

Most children (80%, *n* = 32) concurrently visited 1–3 specialties in the clinic. Of these, 2.5% (*n* = 1), 17.5% (*n* = 7), and 35% (*n* = 14) received physical/occupational therapy on a daily, monthly, and weekly basis, respectively. Overall, 40% (*n* = 16) of the children with CP received physical or occupational therapy in a center or hospital. Almost one-third (30%, *n* = 12) of the children with CP were admitted to the hospital 1–3 times in the past year, and 40% (*n* = 16) were admitted to the PICU 1–3 times in the past year. In addition, approximately 42.5% (*n* = 17) visited the emergency department (ED) 1–3 times in the past year. The details of the results are presented in Table 2.

**Table 2:** Health status and characteristics among children with CP.

Variable	No. (%)
***Child's age (years)***	
<6	13 (32.5)
6–15	24 (60)
>16–20	3 (7.5)
***Child age at diagnosis (months)***	
0–24	31 (77.5)
25–48	5 (12.5)
>48	3 (7.5)
NA	1 (2.5)
***Method of feeding***	
Nasogastric tube	8 (20)
Orally	32 (80)
***Motor skills***	
Able to walk with no issues	10 (25)
Requires wheelchair assistance	14 (35)
Walks with difficulty	13 (32.5)
NA	3 (7.5)
***Activities of daily living***	
Able to perform activities of daily living	5 (12.5)
Able to perform activities of daily living to a degree	6 (15)
Unable to perform activities of daily living	20 (50)
NA	9 (22.5)
***Learning facility attended***	
None	27 (67.5)
Rehabilitation center	6 (15)
School	7 (17)
***No. of days attended in learning facility***	
Daily	11 (27.5)
1–3 per week	4 (10)
NA	25 (62.5)
***No. of clinical specialties visited***	
None	4 (10)
1–3	32 (80)
4–6	4 (10)
***Child receives physical/occupational therapy***	
No	18 (45)
Yes (daily)	1 (2.5)
Yes (weekly)	14 (35)
Yes (monthly)	7 (17.5)
***Location of physical/occupational therapy***	
At home	6 (15)
In a hospital/center	16 (40)
NA	18 (45)

### Depression, anxiety, and stress score-21 (DASS-21)

**Figure 2:**
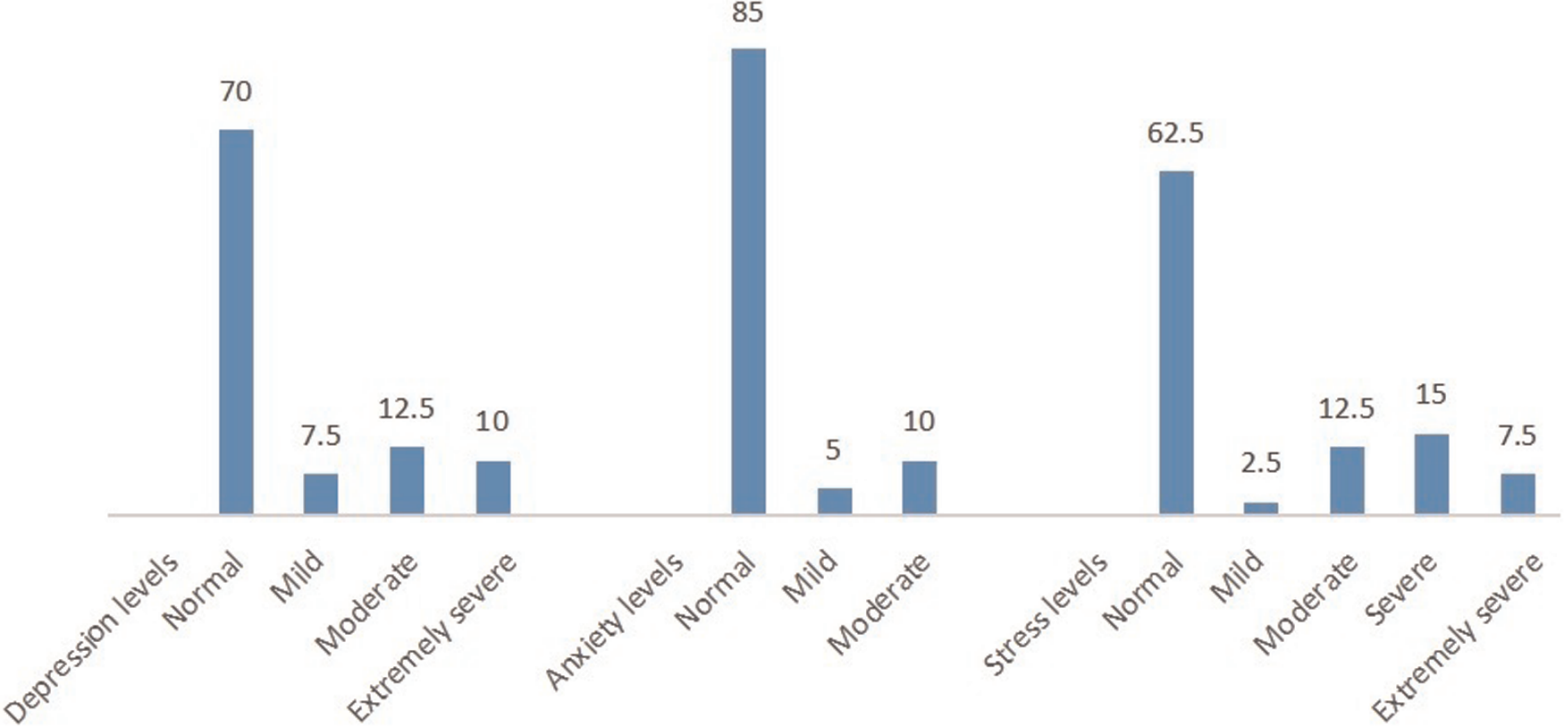
Percentage distribution of the participants according to depression, anxiety and stress status based on the DASS21 scale classification.

According to DASS-21, the median (IQR) depression, anxiety, and stress scores were 4 (10), 4 (11.5), and 10 (21), respectively. Of note, 7.5% (*n* = 3) of the caregivers scored mild depression, 12.5% (*n* = 5) were considered to be impacted with moderate depression, and 10% (*n* = 4) of caregivers reported extremely severe depression according to the scale. Regarding anxiety levels, 5% (*n* = 2) and 10% (*n* = 4) of caregivers reported that they suffered from mild and moderate anxiety, respectively, according to the scale outcomes. In addition, 2.5% (*n* = 1) had mild stress, 12.5% (*n* = 5) had moderate stress, and 15% (*n* = 6) were affected by extremely severe stress, as shown in Figure 2.

An important point to note is that most of the caregivers in our study were mothers (*n* = 29; 72.5%). For mothers’ report, statistical analysis revealed that 14/29 demonstrated normal DASS scores on all parameters. On the other hand, 6/11 fathers had normal DASS scores. Regarding stress scores, we found that 7/29 mothers had reported severe to extremely severe stress according to the scale. In comparison, 4/11 fathers demonstrated moderate to severe scores, and none had extremely severe score. As for anxiety scores, 4/29 mothers had severe to extremely severe scores, while 2/11 fathers had severe to extremely severe anxiety scores. Six out of twenty-nine mothers and 3/11 fathers scored mild to moderate anxiety scores. Furthermore, 4/29 mothers and none of the fathers had extremely severe depression scores. Mild to moderate depression scores were seen in 6/29 mothers, while 2/11 fathers had moderate scores.

As shown in Table 3, significant predictors of extremely severe depression in the caregivers included frequent hospital admission (*p* = 0.004) and ED visits more than six times in the past year (*p* = 0.006). In addition, having 4–6 other children was also significantly associated with moderate and extremely severe depression (*p* = 0.044).

**Table 3:** Relationship between depression status and child health status.

Variable	Depression status				Fisher's Exact test	*p*-value
	Normal No. (%)	Mild No. (%)	Moderate No. (%)	Extremely severe No. (%)		
***Visual impairment***						
I don't know	4 (57.1)	0 (0.0)	3 (42.9)	0 (0.0)	11.65	**0** **.** **033**
Yes	1 (33.3)	0 (0.0)	0 (0.0)	2 (66.7)		
No	23 (76.7)	3 (10)	2 (6.7)	2 (6.7)		
***No. of hospital admissions in the past year***						
None	20 (83.3)	0 (0.0)	2 (8.3)	2 (8.3)	19.24	**0** **.** **004**
1–3	7 (58.3)	3 (25)	2 (16.7)	0 (0.0)		
4–6	1 (50)	0 (0.0)	1 (50)	0 (0.0)		
>6	0 (0.0)	0 (0.0)	0 (0.0)	2 (100)		
***No. of PICU admissions***						
None	14 (73.7)	2 (10.5)	2 (10.5)	1 (5.3)	5.75	0.404
1–3	12 (75)	1 (6.3)	2 (12.5)	1 (6.3)		
4–6	2 (40)	0 (0.0)	1 (20)	2 (40)		
***No. of emergency department visits in the past year***						
None	15 (88.2)	0 (0.0)	1 (5.9)	1 (5.9)	18.17	**0** **.** **006**
1–3	12 (70.6)	2 (11.8)	2 (11.8)	1 (5.9)		
4–6	1 (25)	1 (25)	2 (50)	0 (0.0)		
>6	0 (0.0)	0 (0.0)	0 (0.0)	2 (100)		
***No. of other children***						
None	2 (66.7)	0 (0.0)	1 (33.3)	0 (0.0)	14.21	**0** **.** **044**
1–3	20 (80)	3 (12)	1 (4)	1 (4)		
4–6	3 (33.3)	0 (0.0)	3 (33.3)	3 (33.3)		
7–10	3 (100)	0 (0.0)	0 (0.0)	0 (0.0)		

Bold value: *P*-value < 0.05.

Moreover, caregivers who answered yes when asked if their children had visual impairments were found to have extremely severe depression, contrary to caregivers whose children had normal vision (*p* = 0.033).

Moderate levels of anxiety in the caregivers were significantly associated with children's visual impairments (*p* = 0.041), frequent hospital admissions (*p* = 0.006), PICU admissions of 4–6 times (*p* = 0.006), and ED visits more than six times in the past year (*p* = 0.002). Additionally, caregivers who had 4–6 children also demonstrated moderate levels of anxiety (*p* = 0.007). The details of this are presented in Table 4.

**Table 4:** Relationship between anxiety status and child health status.

Variable	Anxiety status			Fisher's Exact test	*p*-value
	Normal No. (%)	Mild	Moderate		
***Visual impairment***					
I don't know	6 (85.7)	1 (14.3)	0 (0.0)	8.89	**0** **.** **041**
Yes	1 (33.3)	0 (0.0)	2 (66.7)		
No	27 (90)	1 (3.3)	2 (6.7)		
***No. of hospital admissions in the past year***					
None	21 (87.5)	2 (8.3)	1 (4.2)	15.56	**0** **.** **006**
1–3	12 (100)	0 (0.0)	0 (0.0)		
4–6	1 (50)	0 (0.0)	1 (50)		
>6	0 (0.0)	0 (0.0)	2 (100)		
***No. of PICU admissions***					
None	19 (100)	0 (0.0)	0 (0.0)	10.37	**0** **.** **006**
1–3	13 (81.3)	1 (6.3)	2 (12.5)		
4–6	2 (40)	1 (20)	2 (40)		
***No. of emergency department visits in the past year***					
None	16 (94.1)	0 (0.0)	1 (5.9)	16.67	**0** **.** **002**
1–3	16 (94.1)	1 (5.9)	0 (0.0)		
4–6	2 (50)	1 (25)	1 (25)		
>6	0 (0.0)	0 (0.0)	2 (100)		
***No. of other children***					
None	3 (100)	0 (0.0)	0 (0.0)	13.91	**0** **.** **007**
1–3	24 (96)	1 (4)	0 (0.0)		
4–6	4 (44.4)	1 (11.1)	4 (44.4)		
7–10	3 (100)	0 (0.0)	0 (0.0)		

Bold value: *P*-value < 0.05.

There was a significant association between extremely severe stress in caregivers and their children’s visual impairments (*p* = 0.014), frequent hospital admissions (*p* = 0.004), ED visits more than six times a year (*p* = 0.015), and having 4–6 other children (*p* = 0.033) (Table 5).

**Table 5:** Relationship between stress status and child health status.

Variable	Stress status					Fisher's Exact test	*p*-value
	Normal No. (%)	Mild No. (%)	Moderate No. (%)	Severe No. (%)	Extremely severe No. (%)		
***Visual impairment***							
I don't know	2 (28.6)	0 (0.0)	2 (28.6)	3 (42.9)	0 (0.0)	15.94	**0** **.** **014**
Yes	1 (33.3)	0 (0.0)	0 (0.0)	0 (0.0)	2 (66.7)		
No	22 (73.3)	1 (3.3)	3 (10)	3 (10)	1 (3.3)		
***No. of hospital admissions in the past year***							
None	15 (62.5)	0 (0.0)	3 (12.5)	5 (20.8)	1 (4.2)	23.23	**0** **.** **004**
1–3	10 (83.3)	1 (8.3)	1 (8.3)	0 (0.0)	0 (0.0)		
4–6	0 (0.0)	0 (0.0)	1 (50)	1 (50)	0 (0.0)		
>6	0 (0.0)	0 (0.0)	0 (0.0)	0 (0.0)	2 (100)		
***No. of PICU admissions***							
None	15 (78.9)	0 (0.0)	1 (5.3)	2 (10.5)	1 (5.3)	12.02	0.071
1–3	8 (50)	1 (6.3)	4 (25)	3 (18.8)	0 (0.0)		
4–6	2 (40)	0 (0.0)	0 (0.0)	1 (20)	2 (40)		
***No. of emergency department visits in the past year***							
None	12 (70.6)	0 (0.0)	1 (5.9)	3 (17.6)	1 (5.9)	20.33	**0** **.** **015**
1–3	12 (70.6)	0 (0.0)	3 (17.6)	2 (11.8)	0 (0.0)		
4–6	1 (25)	1 (25)	1 (25)	1 (25)	0 (0.0)		
>6	0 (0.0)	0 (0.0)	0 (0.0)	0 (0.0)	2 (100)		
***No. of other children***							
None	2 (66.7)	0 (0.0)	0 (0.0)	1 (33.3)	0 (0.0)	18.93	**0** **.** **033**
1–3	19 (76)	1 (4)	3 (12)	2 (8)	0 (0.0)		
4–6	2 (22.2)	0 (0.0)	1 (11.1)	3 (33.3)	3 (33.3)		
7–10	2 (66.7)	0 (0.0)	1 (33.3)	0 (0.0)	0 (0.0)		

Bold value: *P*-value < 0.05.

## Discussion

This study is one of the few reports evaluating the mental health of caregivers of children diagnosed with CP in Saudi Arabia. One of the major aims of the study was to bring attention to an often neglected aspect of the management of children with CP, which is the mental well-being of their caregivers.

Overall, 15% of the caregivers in our study demonstrated mild to moderate anxiety, which supports the results of other studies showing that caregivers are at an increased risk of developing mental health issues (5, 16). The study outcomes also showed that almost one-third of caregivers had mild to extremely severe depression according to the DASS-21. Moreover, 37.5% of our study population had stress levels ranging from mild to extremely severe. In contrast to the findings of Ramita et al., 2016, in our study, higher scores of caregivers’ stress was significantly associated with children's visual impairment (17). This contradiction in the outcomes may be explained by our small sample size, where only 3 children had visual impairment.

To the best of our knowledge, there is lack of research demonstrating the relationship between children's frequent hospital admissions or ED visits in the past year and their caregivers’ mental health. Factors including hospital admission, ED visits, and their association with caregivers’ depression, stress, and anxiety contribute new evidence to the literature, as no association has been previously studied or hypothesized. The statistical analysis revealed a significant association between the frequency of admission/visits and caregivers’ depression, anxiety, and stress levels. Furthermore, children's mobility did not directly contribute to depression in caregivers in our study, unlike the findings of Gugała et al., 2019, who found that the level of depression was significantly higher in children with impaired mobility, which may be due to the greater number of patients in their study (18). Contrary to the results of a report from Bangalore, India, this study's outcomes revealed no significant association between the level of the child's dependency and the caregivers’ mental health; however, it is important to note the differences in cultures and family bonding patterns between our regions (5). We used the caregiver's marital status as an indicator of family functioning and found no association with mental health, unlike the results of Raina et al., 2005 (4). Unexpectedly, multiple children diagnosed with CP did not reflect the DASS-21 score. In similar line to Raina et al., 2005, socioeconomic factors such as educational level and monthly income were not linked to caregivers’ mental health (4).

Several factors in this study contributed new evidence to the literature. These findings include hospital admission, ED visits, and their association with caregivers’ depression, stress, and anxiety. This study also corroborated earlier findings regarding caregivers of children with CP and the increased risk of developing mental health problems. This study provides one of the first insights into the burdens faced by caregivers of children with CP in Saudi Arabia, the results of which promote the need for healthcare providers and policymakers alike to examine new and existing means of psychosocial support for this group. Importantly, the frequent hospital admissions and ED visits are assumed to have a substantial impact on the caregivers’ well-being. This may cause huge strain on the caregivers as it can result in neglection of other aspects of their lives such as their career and home responsibilities. Another crucial factor is the long-distance travel as some caregivers in this study were residing in other cities. This important concern could be addressed by providing the option of home-healthcare as it could decrease the need to visit the hospital. Thus, enhancing the healthcare for the children as well as the caregivers’ well-being. An additional tool that could provide emotional support and improve the caregivers’ involvement in the community is the introduction of support group services, which are not well-established in our community. Addressing this is of utmost importance as this gives an opportunity for caregivers of children with similar chronic disabling diseases to exchange experiences, feelings about their role as caregivers.

In order to provide holistic care for children with CP and their caregivers, a family-centered approach can be applied. This approach highlights the importance of each family's individuality and how that plays into joint decision making between the family and healthcare providers. The triad of family-centered approach consists of the notions that parents know the most about their children and what is best for them, each family functions in a distinct manner, and family as well as community support provides the child with an environment to function optimally (18, 19) This method has been reported to reduce the rates of depression and distress, resulting in better emotional well-being of caregivers of children with neuro-developmental disorders. This has been clearly illustrated in the work of King et al., where they specifically measured the frequency and severity of depressive symptoms in caregivers including affective, cognitive, and behavioral manifestations, in addition to stress, as indicators of emotional well-being. In which, a family-centered approach was shown to be a strong predictor of caregiver emotional well-being (20).

## Limitations

This study presents few limitations, despite its’ strengths. First, due to cultural differences between ours and other global communities, we believe that mental health issues were not openly disclosed in this study as compared with the Canadian community, which reported a positive GHQ-28 score in more than half of the CP caregivers (4). Second, some variables measured did not show an association with the caregivers’ mental health, which may be due to the limited sample size, which was a result of the limited number of patients with CP in our study setting, NGHA Jeddah, Saudi Arabia. Further multicenter studies are recommended to better illustrate the association between the aforementioned factors and caregivers’ mental health. Finally, online distribution of the questionnaire was a better option than face-to-face interviews because it was more convenient for the caregivers, as many of them lived in rural areas and needed to travel long distances to reach the hospital. However, this posed a problem in the process of completing the questionnaire and may have affected the response rate.

## Conclusion

Caregivers of children with CP exhibited unique mental health challenges that are affected by the child's vision, and the frequent need for acute medical care. The presence of depression, anxiety, and stress in caregivers of children with CP is a serious issue that negatively affects the quality of life. This fact is often neglected by healthcare providers when managing such cases. We aim that this information can be utilized to further improve the care provided to those important figures in the lives of children diagnosed with CP. Healthcare workers should provide early and proactive medical and social support planning for children with CP and their families using a family-centered approach.

## Data Availability

The raw data supporting the conclusions of this article will be made available by the authors, without undue reservation.
