# Supplementary material for: Factors influencing the mental health of caregivers of children with cerebral palsy
Source: Front Pediatr. 2022 Nov 30;10:920744. doi: 10.3389/fped.2022.920744 (PMC9748277; doi:10.3389/fped.2022.920744)
Supplement: Supplementary file 1 [file Table1.docx]

Supplementary Table A: Gross Motor Function Classification System (GMFCS) of Cerebral Palsy Children

| Motor function | No. (%) | Corresponding GMFCS |
| --- | --- | --- |
| Able to walk with no issues | 10 (25) | Level I |
| Walks with difficulty | 13 (32.5) | Level II |
| Requires wheelchair assistance | 14 (35) | Level III-IV |
| Can’t move | 3 (7.5) | Level V |
